# Adaptation to fluctuations in temperature by nine species of bacteria

**DOI:** 10.1002/ece3.3823

**Published:** 2018-02-14

**Authors:** Kati Saarinen, Jouni Laakso, Leena Lindström, Tarmo Ketola

**Affiliations:** ^1^ Department of Biological and Environmental Science Centre of Excellence in Biological Interactions University of Jyväskylä Jyväskylä Finland; ^2^ Department of Biological and Environmental Science Centre of Excellence in Biological Interactions University of Helsinki Helsinki Finland

**Keywords:** experimental evolution, reaction norm, temperature fluctuation, tolerance curve

## Abstract

Rapid environmental fluctuations are ubiquitous in the wild, yet majority of experimental studies mostly consider effects of slow fluctuations on organism. To test the evolutionary consequences of fast fluctuations, we conducted nine independent experimental evolution experiments with bacteria. Experimental conditions were same for all species, and we allowed them to evolve either in fluctuating temperature alternating rapidly between 20°C and 40°C or at constant 30°C temperature. After experimental evolution, we tested the performance of the clones in both rapid fluctuation and in constant environments (20°C, 30°C and 40°C). Results from experiments on these nine species were combined meta‐analytically. We found that overall the clones evolved in the fluctuating environment had evolved better efficiency in tolerating fluctuations (i.e., they had higher yield in fluctuating conditions) than the clones evolved in the constant environment. However, we did not find any evidence that fluctuation‐adapted clones would have evolved better tolerance to any measured constant environments (20°C, 30°C, and 40°C). Our results back up recent empirical findings reporting that it is hard to predict adaptations to fast fluctuations using tolerance curves.

## 1 INTRODUCTION

Among the physical environmental variables, temperature has been called “a major driving force in evolution” (Hochachka & Somero, 2002). Evolution of temperature tolerance in general, and adaptation to high or increasing temperatures in particular, has been studied rather widely (reviewed in: Araújo et al., 2013; Hoffmann & Sgro, 2011).

The demand for understanding consequences of especially fluctuating environments has grown bigger as climate change scenarios predict increased fluctuations in temperature and other environmental conditions (Stocker et al., 2014).

The most traditional way of testing the tolerance of species or genotypes to environmental variation, like temperature, is by depicting species performance across different constant environments using tolerance curves (Huey & Kingsolver, 1989, 1993). For example, broad/flat tolerance curves (superior tolerance of extreme temperatures at both ends of the curve) and high elevation of the tolerance curve (superior tolerance of all the experienced temperatures) could be predictors of good tolerance to temperature fluctuations (Scheiner & Yampolsky, 1998). There is experimental evidence to show that constant environments favor specialism, and fluctuating or heterogeneous environments select for genotypes that are capable of tolerating a wide range of conditions (Condon, Cooper, Yeaman, & Angilletta, 2014; Duncan, Fellous, Quillery, & Kaltz, 2011; Kassen, 2002; Ketola et al., 2013; Venail, Kaltz, Olivieri, Pommier, & Mouquet, 2011).

However, not all mechanisms on adaptation to fluctuating temperatures might be captured in tolerance curves measured at constant temperatures. For example, reversible phenotypic plasticity (Bennett & Hughes, 2009; Hughes, Cullum, & Bennett, 2007), via increased heat shock protein expression at extremes (Ketola, Laakso, Kaitala, & Airaksinen, 2004; Sørensen, Kristensen, & Loeschcke, 2003), or increased ability to utilize the short time window of optimal conditions between the extremes (Gilchrist, 1995; New et al., 2014), can be difficult to observe from tolerance curves (Ketola, Kellermann, Loeschcke, Lopez‐Sepulcre, & Kristensen, 2014). Evolution could also lead to bet‐hedging, in which an individual expresses different phenotypes with a certain probability, in completely random environments (Arnoldini, Mostowy, Bonhoeffer, & Ackermann, 2012; King & Masel, 2007). Therefore, to test the level of adaptation to fluctuations, we should preferably estimate fitness in fluctuating environments, rather than deducing it via tolerance curves (Ketola & Kristensen, 2017; Ketola & Saarinen, 2015; Ketola et al., 2014; Schulte, Healy, & Fangue, 2011; Sinclair et al., 2016).

Experimental evolution studies are efficient systems for testing emergence of adaptations to various kinds of selection pressures (reviewed by Buckling, Maclean, Brockhurst, & Colegrave, 2009; Kawecki et al., 2012) and not surprisingly there exists quite a large body of the literature on evolution in fluctuating environments. However, most of the studies concentrate on changes in tolerance curves as a response to fluctuations (reviewed by Kassen, 2002), rather than testing directly if tolerance to fluctuations has increased as a consequence of selection. So far, only a handful of studies have actually tested performance in fluctuating environments (Hughes et al., 2007; Kassen & Bell, 1998; Ketola & Saarinen, 2015; Leroi, Lenski, & Bennett, 1994; Magalhaes, Cailleau, Blanchet, & Olivieri, 2014; this study). However, most of the experiments consider mostly very slow fluctuations and thus fresh work on faster frequencies of fluctuations is direly needed.

**Figure 1:**
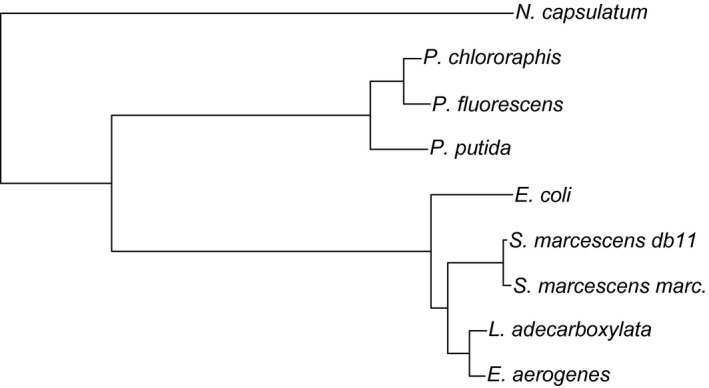
Phylogeny of the study species based on 16S rRNA. The scale bar represents the number of nucleotide substitutions per site. The tree includes the sequences FJ971882 (*Enterobacter aerogenes*), GQ856082 (*Leclercia adecarboxylata*), NR_041980 (*Serratia marcescens* ssp*. marcescens*), HG326223 (*Serratia marcescens* ssp. DB11 [whole genome, 16S rRNA part included]), NR_024570 (*Escherichia coli*), AF094736 (*Pseudomonas putida*), AF094725 (*Pseudomonas fluorescens*), AB680102 (*Pseudomonas chlororaphis*), and NR_025838 (*Novosophingobium capsulatum*). The sequence accession numbers were obtained from the NCBI nucleotide sequences database

We ran parallel experimental evolution studies with nine different species/subspecies of bacteria (instead of concentrating on one species Figure 1) to create clones adapted to either fluctuating (20°C, 30°C, 40°C, at 2‐hr intervals) or constant (30°C) temperature. After the experimental evolution, these bacterial clones were first tested for their ability to tolerate fluctuating temperature in fluctuating conditions. If fluctuating adapted clones perform better at fluctuating conditions, we can then suggest that these clones have indeed adapted to tolerate fluctuations better. Then, we measured temperature tolerance in a few constant temperatures, to reveal if evolution had led to changes in tolerance in constant environments (20°C; 30°C; 40°C). With these data, we tested the generality of the idea that fluctuations should select genotypes that are good at tolerating fluctuating environments and if adaptation to fluctuations could be predicted from some of the measurements taken in constant environments. By replicating whole experimental evolution experiment with nine species allows us to generalize results much better than results from normal single species experimental evolution study.

## 2 MATERIALS AND METHODS

### 2.1 Study species

**Figure 2:**
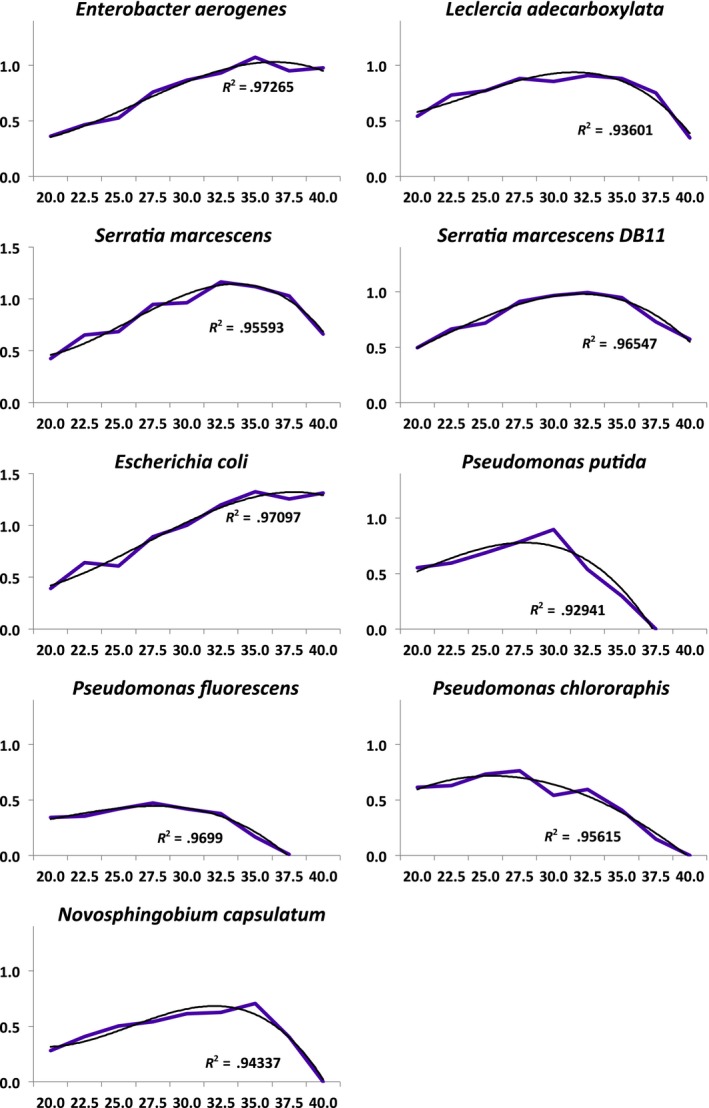
Measured thermal tolerance of the study species (°C) expressed as maximum growth rate (OD 600 nm/hr) (Pink line: measurements, black line: third degree polynomial fitted to the measurement data)

We used nine different, well‐known and easily‐culturable bacteria (eight different species and one subspecies) in the experiment. All the species, except *Serratia marcescens* ssp. DB11 (Flyg, Kenne, & Boman, 1980), were originally obtained from ATCC® (American Type Culture Collection) and stored at −80°C: *Enterobacter aerogenes* ATCC® 13048™, *Leclercia adecarboxylata* ATCC® 23216™, *Serratia marcescens* ssp. *marcescens* ATCC® 13880™, *Escherichia coli* ATCC® 11775™, *Pseudomonas putida* ATCC® 12633™, *Pseudomonas fluorescens* ATCC® 13525™, *Pseudomonas chlororaphis* ATCC® 17418™ and *Novosphingobium capsulatum* ATCC® 14666™. The species were chosen based on their abilities to grow well in the same medium and to tolerate the rapidly fluctuating temperature range (20°C, 30°C, 40°C, temperature change at 2‐hr intervals). All species had shorter minimal generation time than experimental fluctuation. Measured from ancestors and in NB (values reflect minimum generation time found at optimal conditions and at 30°C (experimental mean temperature) for each of the species. *Enterobacter aerogenes* (optimum: 0.647 hr, at +30°C: 0.801 hr), *Leclercia adecarboxylata* (optimum: 0.762 hr, at +30°C: 0.811 hr), *Serratia marcescens* ssp*. marcescens* (optimum: 0.594 hr, at +30°C: 0.720 hr), *Serratia marcescens db11* (optimum: 0.699 hr, at +30°C: 0.718 hr), *Escherichia coli* (optimum: 0.523 hr, at +30°C: 0.692 hr), *Pseudomonas putida* (optimum: 0.774 hr, at +30°C: 0.774 hr), *Pseudomonas fluorescens* (optimum: 1.468 hr, at +30°C: 1.652 hr), *Pseudomonas chlororaphis* (optimum: 0.910 hr, at +30°C: 0.910 hr), and *Novosphingobium capsulatum* (optimum: 0.984 hr, at +30°C: 1.131 hr). The species have different optimum temperatures and species broadly fall into two categories based on their performance at extreme conditions: Three *Pseudomonas* species and *N. capsulatum* can tolerate +40°C only short periods of time, where as other species can grow well at +40°C (Figure 2).

### 2.2 Evolution treatment

To create bacterial strains that were either adapted to constant (30°C) or fluctuating (20°C, 30°C, 40°C) temperatures, we performed a 79‐day‐long evolution treatment. We reared 10 populations of each study species both in constant and in rapidly fluctuating temperature regimes (90 populations in both treatments, 180 populations in total). We started the experimental populations from single bacterial colonies growing on nutrient agar. For each species, a single colony was transferred to separate 10 ml centrifuge tubes containing 1 ml of nutrient broth. The bacteria were propagated for 3 days at 30°C to obtain high density. After this, each culture was divided into 10 wells of a 100‐well Bioscreen C® (Growth curves Ltd, Helsinki, Finland) spectrophotometer plate (40 μl of bacteria inoculum into 400 μl of nutrient broth (Nutrient broth: 10 g of nutrient broth [Difco, Becton & Dickinson, Sparks, MD, USA] and 1.25 g of yeast extract [Difco] in 1 L of dH_2_O)) per well; each species in separate plates and again propagated for 3 days to high density. From these 10 replicates for each species, we initiated the treatments in two thermal cabinets (ILP‐12; Jeio Tech, Seoul, Korea): 10 populations of each nine bacterial species in each cabinet. From the same 10 replicates, we also stored the ancestors in cryotubes at −80°C. As the populations were founded from single colonies, the starting genetic variance among the populations within species is assumed to be close to zero. The two different temperature treatments were constant 30°C and fluctuating 2 hr 20°C, 2 hr 30°C, 2 hr 40°C. We chose this temperature range to induce as severe thermal stress as possible without causing extinctions. The bacterial populations were transferred into new wells of the spectrophotometer plates every third day. Three days correspond to a minimum of 3.32 generations in all the species and treatments (Bennett, Lenski, & Mittler, 1992) and are the same for all species. The evolution treatment was continued for 79 days (theoretically ca. 86 generations, Bennett et al., 1992). Twice a month, populations were transferred between the chambers in order to prevent cabin effects from interfering with the evolutionary treatment effects. Samples from each population were stored at −80°C (1:1 high‐density bacterial population in nutrient broth and 80% glycerol) twice a month.

### 2.3 Extraction of bacterial clones after the experiment

After the evolution treatments, bacterial clones were extracted from populations with dilution plate technique. Two dilution plates (10^6^ dilution) for each population were first propagated for several days at +30°C depending on how long it took for the colonies to grow big enough for further sampling. From these plates, we randomly chose four clones from each experimental population and from each species. Note that within every species, the procedures were the same for both evolutionary treatments. The selected 720 clones were propagated in the medium (300 μl of nutrient broth in 1.5 ml Eppendorf tubes) for 24 hr at 30°C (except 3 days for *N. capsulatum*) to ensure they had reached high enough density. The clones, each mixed with 80% glycerol (1:1), were then pipetted to spectrophotometer plates in prerandomized order, and the plates were frozen to −80°C for further use. The use of a cryoreplication system (Duetz et al., 2000) with clone libraries allows efficient workflow without thawing the strains, with randomized and balanced settings for each species that are easy to use for growth measurements numerous times.

### 2.4 Growth measurements

To evaluate whether the constant and fluctuating evolution treatments caused differences between the bacterial clones of each species, we measured growth of the clones at fluctuating (1 hr 20°C – 1 hr 40°C) and constant (20°C, 30°C, 40°C) temperatures. Each measurement was initiated by cryoreplicating clones from frozen plates to plates containing fresh medium, with cryoreplicator system described in Duetz et al. (2000). To standardize growth conditions and to get rid of glycerol residues, the clones grew 3 days at 30°C after which the 40 μl of bacteria solution was pipetted on new Bioscreen plate, filled with fresh NB. The growth measurements were performed in temperature‐controlled spectrophotometers (Bioscreen C®, Oy Growth Curves Ab, Ltd, Helsinki, Finland), where one can adjust temperatures while measuring growth. Utilizing this property, we were able to fluctuate temperatures and follow instant changes in biomass. The fluctuation in this part of the experiment was faster than what the strains experienced during the evolution treatment, to allow capture of evolutionary effects on maximal instantaneous growth rate under fluctuations. The optical densities (OD) of the colonies were recorded at 600 nm absorbance and 5‐min intervals for 3–5 days, until the growth in all wells had ceased. The length of the measurement depended on the temperature used and the growth rate of the study species.

The raw growth measurement data were first processed using script written in MATLAB (MATLAB R2010b, The MathWorks Inc., Natick, MA, USA). The script was used to determine maximum growth rate and biomass yield values for each bacterial clone. From log‐transformed data, the script finds the time of fastest growth by fitting linear regressions of time and log (OD) on a 25‐time step (125 min) sliding window. The fastest growth rate equals steepest slope of linear regression found in sliding windows (log transformation linearizes the exponential growth). The yield corresponds to the largest average population size (OD) found from sliding windows. Growth rate indicates the speed of resource utilization during growth in the batch culture and yield allows deduction in the amount of biomass a species or clone can produce with given resources. Both of the traits are beneficial for bacterial fitness (see: Ketola & Saarinen, 2015).

### 2.5 Data‐analysis

To explore whether fluctuations selected for tolerance to thermal fluctuations, we modeled the data with linear mixed model (REML) for each species separately. We used maximum growth rate and biomass yield as dependent variables with evolution treatment as a fixed effect (SPSS v. 20, IBM). The models contained population as a random effect to control for the nonindependency of clones extracted from the same replicate populations. This effect was nested within the evolutionary treatments. In addition, inoculum size (OD of the inoculum) was fitted as a covariate to control for the differences in starting cell densities. We ran these models for each species for all measurement temperatures (fluctuating, 20°C, 30°C, 40°C).

These single species analyses were combined meta‐analytically to handle and measure heterogeneity of the effect sizes and to incorporate phylogenetic dependency of observations in a random effect meta‐analysis with metaphor R‐package (Viechtbauer, 2010). The effect sizes are based on *t* test on estimated marginal means from species and temperature specific models, for testing whether two evolutionary treatments differ in their growth or yield (Table 1).

**Table 1:** Results of pairwise tests exploring if clones adapted to fluctuating or constant temperature have higher yield or growth in different environments

Environment	Species	Yield	*SE*	*p*	Growth rate	*SE*	*p*
Fluctuating	*N. capsulatum*	0.083	.042	.053	0.053	.026	.048
Fluctuating	*P. chlororaphis*	0.024	.042	.569	−0.018	.026	.49
Fluctuating	*P. fluorescens*	0.489	.043	.001	0.017	.027	.525
Fluctuating	*P. putida*	0.114	.043	.009	0.082	.027	.003
Fluctuating	*S. marcescens marc*.	−0.011	.03	.699	0.034	.043	.426
Fluctuating	*S. marcescens db11*	0.016	.03	.587	0.061	.043	.161
Fluctuating	*E. coli*	0.052	.03	.085	0.075	.043	.088
Fluctuating	*E. aerogenes*	−0.018	.03	.559	−0.066	.043	.133
Fluctuating	*L. adecarboxylata*	0.049	.03	.103	−0.043	.043	.32
20°C	*N. capsulatum*	0.002	.043	.967	0.006	.021	.768
20°C	*P. chlororaphis*	−0.1	.042	.02	0.012	.021	.57
20°C	*P. fluorescens*	−0.013	.045	.769	−0.01	.022	.669
20°C	*P. putida*	−0.001	.043	.973	−0.129	.021	.001
20°C	*S. marcescens marc*.	−0.006	.054	.918	0.014	.02	.481
20°C	*S. marcescens db11*	0.081	.054	.139	−0.007	.02	.732
20°C	*E. coli*	0.067	.055	.225	0.004	.02	.831
20°C	*E. aerogenes*	−0.059	.056	.291	0	.021	.993
20°C	*L. adecarboxylata*	0.02	.054	.714	0.007	.02	.727
30°C	*N. capsulatum*	0.006	.056	.92	0.01	.034	.772
30°C	*P. chlororaphis*	−0.057	.055	.303	−0.034	.033	.311
30°C	*P. fluorescens*	0.024	.056	.669	−0.073	.034	.036
30°C	*P. putida*	0.065	.055	.241	−0.148	.033	.001
30°C	*S. marcescens marc*.	0.067	.048	.17	−0.003	.026	.914
30°C	*S. marcescens db11*	0.031	.049	.534	−0.004	.026	.884
30°C	*E. coli*	0.007	.049	.895	−0.035	.027	.185
30°C	*E. aerogenes*	−0.027	.049	.579	−0.063	.026	.019
30°C	*L. adecarboxylata*	0.015	.048	.755	−0.054	.026	.041
40°C	*N. capsulatum*	0.07	.029	.022	0.023	.015	.131
40°C	*P. chlororaphis*	−0.006	.042	.887	−0.022	.02	.272
40°C	*P. fluorescens*	0.021	.072	.775	0.031	.031	.328
40°C	*S. marcescens marc*.	0.009	.049	.859	0.032	.037	.385
40°C	*S. marcescens db11*	0.014	.047	.763	0.021	.035	.559
40°C	*E. coli*	0.08	.048	.096	−0.001	.035	.984
40°C	*E. aerogenes*	−0.048	.048	.321	0.008	.036	.818
40°C	*L. adecarboxylata*	0.027	.055	.622	0.005	.041	.911

Positive estimate for difference indicates that the fluctuation‐adapted clones have a higher yield or growth rate than constant (30°C)‐adapted clones. Values indicate estimated marginal means from mixed models testing for the fixed effect of evolution, and random effect of population, nested within evolutionary treatment. All models also included inoculum size as a continuous covariate to control for different starting densities in growth measurements (not shown). These results were compiled in the meta‐analysis.

The significance of phylogenetic effect using 16sRNA‐based phylogeny (Figure 1) was assessed with likelihood ratio tests. Hence, none of the trait indicated improved model fit with phylogenetic information (LRT non significant), we conclude that our results are not sensitive to phylogenetic nonindependence, and we present data from random effect models without phylogenetic effects. It is noteworthy that our initial aim was not to test phylogenetic effects in the first place, as that would require larger dataset. Similarly nine species is still rather small sample size in hand for fitting effects of species differences for explaining differences in evolution. Species causing heterogeneity in analysis were removed from the final analyses (Table 2A,B). Moreover, *Pseudomonas putida* did not grow at constant 40°C (Figure 1).

**Table 2:** Results of random effect meta‐analysis testing whether the fluctuation‐adapted clones outperform (positive estimate) or underperform (negative estimates) the clones adapted to constant 30°C. Panel A denotes analyses without species causing heterogeneity in meta‐analysis. Panel B contains analysis results with all species. In both panels, *Q* stands for heterogeneity statistics and probability associated with it denotes its significance

Environment	Trait	Estimate	*SE*	*z*	*p*	*Q*	*p*	Notes
(A)								
Fluctuating 20–40°C	Yield	0.415	.1619	2.5635	**.0104**	8.1601	.3187	*P. fluorescens* omitted^a^
Fluctuating 20–40°C	Growth rate	0.1743	.1612	1.0815	.2795	10.9911	.139	*P. putida* omitted^a^
Constant 20°C	Yield	−0.0377	.1964	−0.192	.8477	9.0592	.3373	
Constant 20°C	Growth rate	0.0694	.1584	0.4384	.6611	1.0737	.9935	*P. putida* omitted^a^
Constant 30°C	Yield	0.1224	.1501	0.8158	.4146	4.175	.841	
Constant 30°C	Growth rate	−0.5802	.1551	−3.7416	**.0002**	13.3459	.1005	
Constant 40°C	Yield	0.2109	.1602	1.3164	.1881	6.7024	.4605	
Constant 40°C	Growth rate	0.1699	.1594	1.0659	.2864	3.9484	.7857	
(B) All species in the model								
Fluctuating 20–40°C	Yield	0.5575	.1593	3.5003	**.0005**	32.3929	<.0001	
Fluctuating 20–40°C	Growth rate	0.2835	.1532	1.8502	.0643	15.7208	.0466	
Constant 20°C	Yield	−0.0377	.1964	−0.192	.8477	9.0592	.3373	
Constant 20°C	Growth rate	−0.1008	.1534	−0.6572	.511	19.39	.0129	
Constant 30°C	Yield	0.1224	.1501	0.8158	.4146	4.175	.841	
Constant 30°C	Growth rate	−0.5802	.1551	−3.7416	**.0002**	13.3459	.1005	
Constant 40°C	Yield	0.2109	.1602	1.3164	.1881	6.7024	.4605	
Constant 40°C	Growth rate	0.1699	.1594	1.0659	.2864	3.9484	.7857	

a Due to heterogeneity. bold values indicate significant effect

## 3 RESULTS

**Figure 3:**
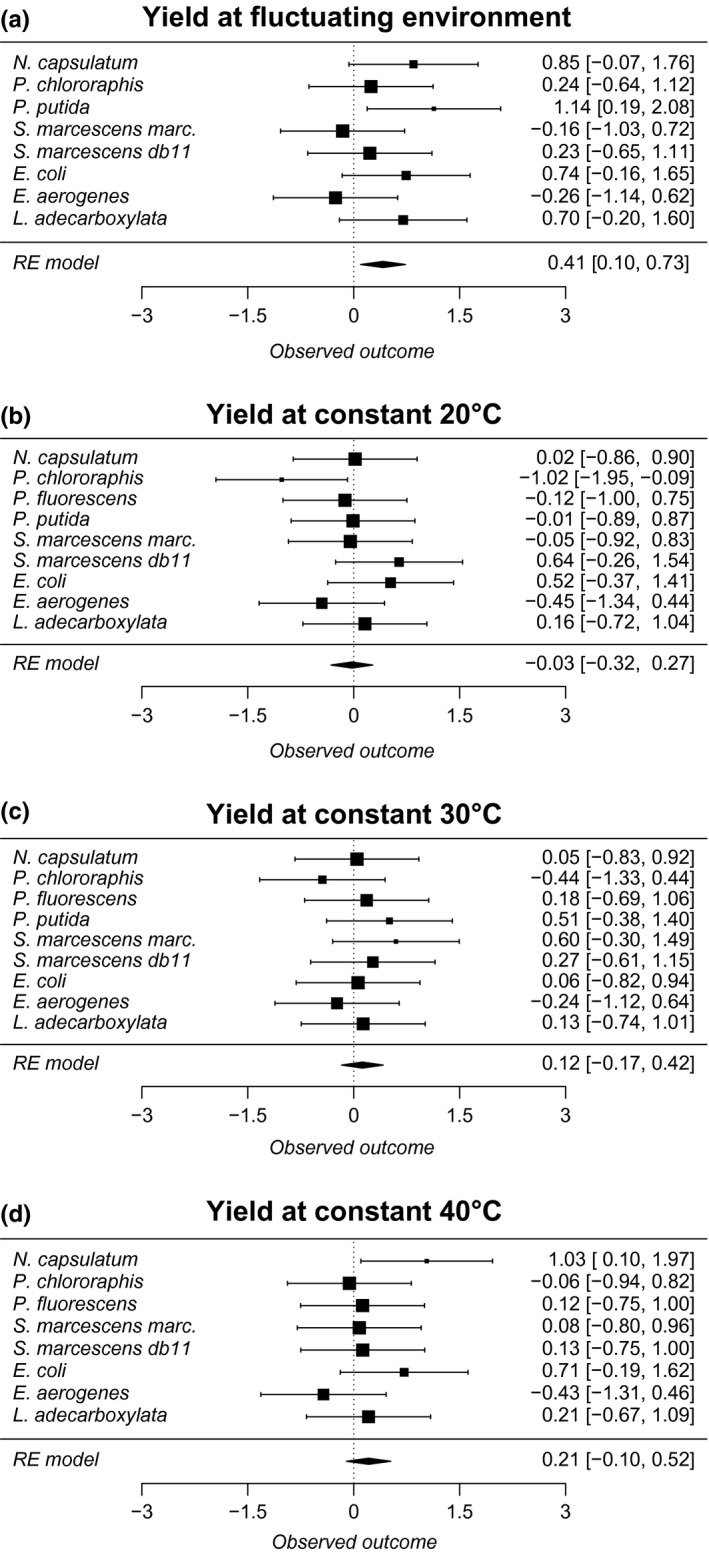
Forest plots of meta‐analyses (corresponds to Table 2) of the biomass yield in the four different measurement temperatures (a) fluctuating (2 hr 20°C, 2 hr 30°C, 2 hr 40°C) (b) constant 20°C (c) constant 30°C (d) constant 40°C for all studied species. If effect sizes are higher than zero, it indicates a better performance of clones adapted to fluctuating temperature than clones adapted to constant (30°C) temperature. Effect sizes and their confidence intervals (±95%) are denoted in the right‐hand side of the figure. RE model indicates estimate for random effect meta‐analysis model. Different sized symbols denote the magnitude of weighing (larger more weight, smaller less)

**Figure 4:**
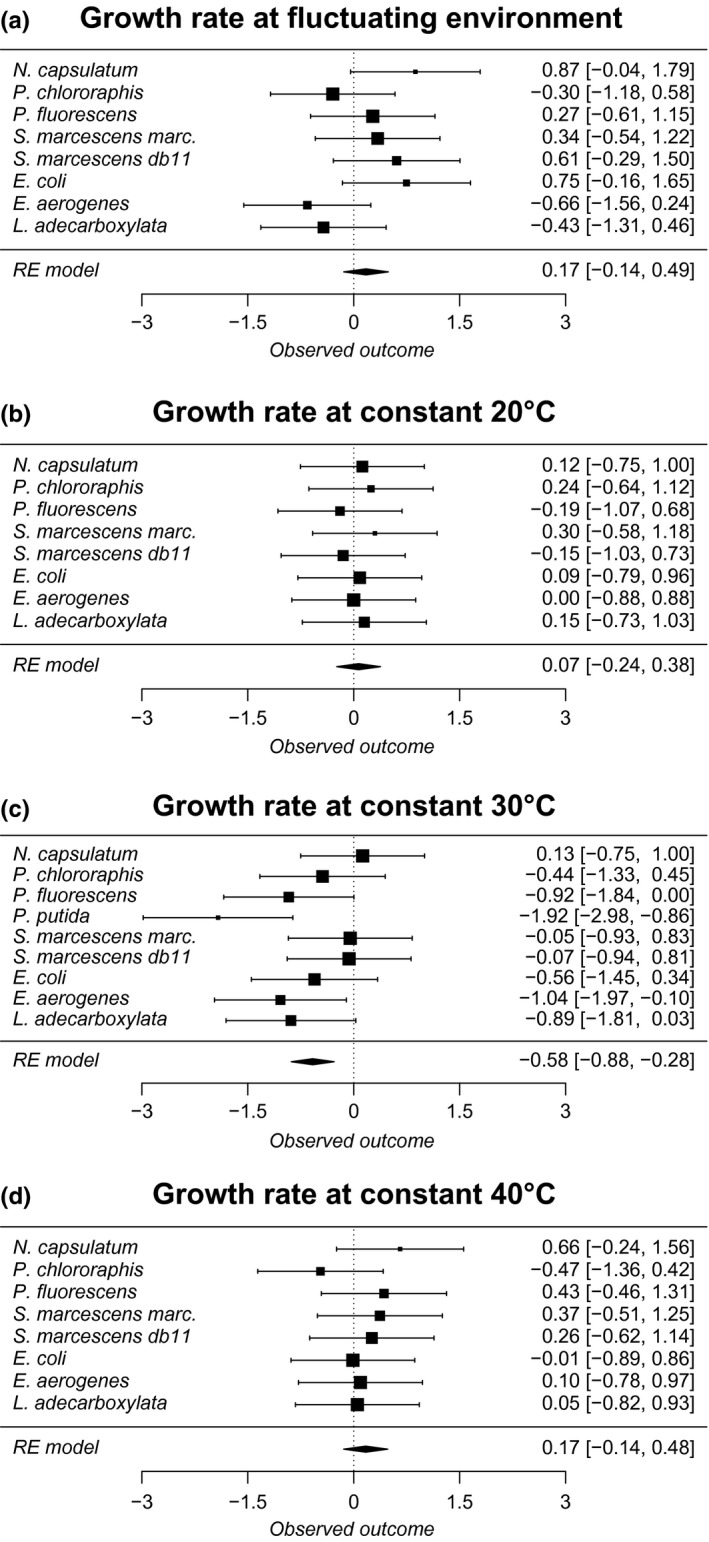
Forest plots of meta‐analyses (corresponds to Table 2) of the growth rate in different measurement temperatures (a) fluctuating (2 hr 20°C, 2 hr 30°C, 2 hr 40°C) (b) constant 20°C (c) constant 30°C (d) constant 40°C for all studied species. If effect sizes are higher than zero, it indicates a better performance of clones adapted to fluctuating temperature than clones adapted to constant (30°C) temperature. Effect sizes and their confidence intervals (±95%) are denoted in the right‐hand side of the figure. RE model indicates estimate for random effect meta‐analysis model. Different sized symbols denote weighing (larger more weight, smaller less)

The raw data for pairwise tests exploring whether clones adapted to fluctuating or constant temperature have higher yield or growth in different environments are shown in Table 1. These data were used for meta‐analysis, which confirmed that overall the clones that had evolved in fluctuating environment were able to produce higher biomass yield in fluctuating environment than clones that evolved in constant environment (Table 2A, Figure 3). However, there were no differences in the maximum growth rate between clones in fluctuating conditions. When clones were assessed in constant conditions, the only difference between the evolution treatments was that the clones that had evolved at constant 30°C had better growth rate at constant 30°C (Table 2A, Figure 4) than the clones evolved in fluctuating environment.

One plausible explanation for our results could be that different temperatures could be more critical to different species due to their different thermal optima. We further tested this by dividing the data into cold‐adapted (three *Pseudomonas* species and *N. capsulatum*) and hot‐adapted (all the rest) species (Figure 1) and tested whether different traits or hot and cold adaptation regarding thermal optima would indicate evolution of thermal tolerance in constant temperatures. However, these analyses indicated no evidence for adaptation to fluctuating environment (Table 3).

**Table 3:** Meta‐analysis exploring if hot‐ and cold‐adapted species express their evolutionary changes in different traits measured in hot or cold constant environments. Cold adapted species refers to *N. capsulatum* and *Pseudomonas* species. All the rest are considered hot adapted

Cold adapted species		Hot adapted species		Estimate	*SE*	z	*p*	*Q*	*p*
Environment	Trait	Environment	Trait						
Constant 40°C	Yield	Constant 20°C	Yield	0.2267	.1604	1.4132	0.1576	7.2228	.4061
Constant 40°C	Growth rate	Constant 20°C	Growth rate	0.1225	.1593	0.769	0.4419	4.136	.7639
Constant 40°C	Yield	Constant 20°C	Growth rate	0.1727	.1595	1.0827	0.279	4.3442	.7394
Constant 40°C	Growth rate	Constant 20°C	Yield	0.1758	.1602	1.0972	0.2725	7.2277	.4056

## 4 DISCUSSION

We exposed several species of bacteria to fluctuating or constant temperature for 2.5 months and found that overall fluctuation‐adapted bacterial clones were able to attain higher yield at fluctuating temperature than strains evolved in constant environment (Figure 3), indicating clear adaptation to fluctuations in temperature. These results indicate that the fluctuating conditions could select for efficient resource use. Interestingly, previous study performed with *S. marcescens* in even faster temperature fluctuation indicated an increased growth rate during fluctuations (Ketola & Saarinen, 2015). This suggests that the speed of fluctuations could select for different mechanisms such as efficient resource use or adapting to grow quickly to make most of the fast changes in environment. However, it is noteworthy that many other aspects of these experiments were different, such as renewal rate and the medium used, making the comparison of these studies problematic. Such comparison problems also pinpoint the reasoning why repeating studies with several species in similar settings is very important. Multispecies studies are also efficient of reducing file drawer effects that hamper conventional research synthesis based on separate publications.

Interestingly we did not find any evidence that the measurable adaptation to fluctuating conditions could be deduced from the improved yield measurements in the constant temperatures (20°C; 30°C; 40°C). This is contrary to the theoretical expectations, as the tolerance measured in constant environments has been considered indicative of the level of adaptation to fluctuating environments (Dobzhansky & Spassky, 1963; Duncan et al., 2011; Gilchrist, 1995; Kassen, 2002; Ketola et al., 2013; Levins, 1968; Venail et al., 2011). The fluctuation‐adapted clones had lower growth rate than the constant‐adapted clones at constant 30°C which was the average temperature during the experiment, reflecting the results found in Ketola and Saarinen (2015). It could be that same species showing improved tolerance to fluctuations could trade‐off their evolved capability to stand fluctuations by having lower growth rate at constant 30°C. However, we did not find statistical support for this idea from analysis exploring yield at 30°C with growth rate at 30°C as a covariate (est: −0.2717, *SE* = .2812, *z* = −0.9660, *p* = .3340).

Naturally three assessed temperatures is a small number for fitting actual tolerance curves. However, if we would expect to see changes in growth and yield, these three temperatures (20°C, 30°C, or 40°C) should capture the difference as they match the temperatures experienced during the experimental evolution. Furthermore, fine‐tuning the temperature curves with an addition of measurements in constant temperatures within the “transition phase” temperatures do not necessary reveal the adaptation to fluctuating conditions either (see results of Ketola & Saarinen, 2015 for *S. marcescens*).

One plausible explanation for our results could be that different temperatures could be more critical to different species due to their different thermal optima, complicating finding the universal evolutionary effects from constant measurement temperatures. To test this idea further, we classified the data into two groups: cold‐adapted (three *Pseudomonas* species and *N. capsulatum*) and hot‐adapted (all the rest) species (Figure 4). After this, we used data from cold temperatures and hot temperatures, and from growth rate and yield to test whether hot‐adapted species evolve better cold tolerance and cold‐adapted species evolve better hot tolerance. However, these analyses, where we “cherry pick” data from different parts of the tolerance range, and different traits (growth and yield), indicated no evidence for adaptation to fluctuating environment (Table 3). Moreover, when individual species results (Table 1) are followed, it is also evident that only one species (*N. capsulatum*) indicate significant improvement of yield at 40°C if clones had evolved in fluctuating conditions. All other significant tests from constant conditions indicate the opposite: Fluctuation‐adapted strains do worse in constant conditions (Table 1). Thus, it is clear that fast temperature fluctuations do not cause observable benefits when growth traits are measured in these constant conditions.

Slow fluctuations have been found to select for faster growth and higher yield when measured in constant conditions (Ketola et al., 2013), which contrasts to our findings. It could be that adaptations to chronic, days long, exposures can be predicted from the tolerance curves, whereas fast, hourly and acute (as in Ketola & Saarinen, 2015; and here), fluctuations are more visible in traits that are linked with short exposures to extreme temperatures, like expression of heat shock proteins (HSP's) (Ketola et al., 2004; Sørensen et al., 2003). Yet, experimental evolution studies on adaptation to fluctuating environments are numerous (Kassen, 2002), only a few experimental evolution studies have measured performance at both the constant and fluctuating environments and very few have studied further the possible mechanisms. Only one study suggests a positive association between tolerating constant and fluctuating environments (Hughes et al., 2007), and the majority of studies show either no clear association (Bennett & Lenski, 1993; Kassen & Bell, 1998; Ketola et al., 2004; Leroi et al., 1994) or that adaptations to tolerate fluctuating temperatures trades‐off with tolerating constant temperatures (Ketola & Saarinen, 2015; New et al., 2014). These few studies and our data presented here thus indicate that tolerating constant conditions might have little in common with tolerating fluctuating environments or even may be competing from shared resources. This warrants attention when reaction norms or tolerance curves are used to judge genotypes or species for their ability to tolerate fluctuations in animal and plant breeding as well as in conservation biology (see also Ketola & Kristensen, 2017; Schulte et al., 2011; Sinclair et al., 2016).

In our experiment, where nine bacterial species were grown independently in constant or rapidly fluctuating environments, we found that fluctuations increased species’ tolerance to fast fluctuations. In addition, our results give support to the idea that tolerances measured in constant environments might fail to capture adaptations to fast fluctuations (Ketola & Saarinen, 2015; Ketola et al., 2014). Effects of adaptation mechanisms, some of which might not be captured in tolerance curves, are important to be taken into account in predicting species’ or genotypes’ ability to survive climate change associated environmental fluctuations. By this experiment, we are also able to show that the evolutionary effects were observable over several species, using meta‐analysis. Something that is not possible with single species studies.

## CONFLICT OF INTEREST

The authors declare no conflict of interest.

## AUTHORS CONTRIBUTION

TK and KS designed the experiment, analyzed the data, and wrote first versions of the manuscript. All finalized the manuscript and agreed on the contents of the current version.
